# Response of glyphosate-resistant and susceptible biotypes of *Echinochloa colona* to low doses of glyphosate in different soil moisture conditions

**DOI:** 10.1371/journal.pone.0233428

**Published:** 2020-05-20

**Authors:** Mahboobeh Mollaee, Amar Matloob, Ahmadreza Mobli, Michael Thompson, Bhagirath Singh Chauhan

**Affiliations:** 1 Department of Agrotechnology, Faculty of Agriculture, Ferdowsi University of Mashhad, Mashhad, Iran; 2 The Centre for Crop Science, Queensland Alliance for Agriculture and Food Innovation (QAAFI) and School of Agriculture and Food Sciences (SAFS), The University of Queensland, Gatton, Queensland, Australia; 3 Department of Agronomy, Muhammad Nawaz Shareef University of Agriculture, Multan, Pakistan; University of Agriculture, PAKISTAN

## Abstract

To evaluate the hormetic effect of glyphosate on *Echinochloa colona*, two pot studies were done in the screenhouse at the Gatton Campus, the University of Queensland, Australia. Glyphosate was sprayed at the 3–4 leaf stage using different doses [(0, 5, 10, 20, 40, 80 and 800 g a.e. ha^-1^) and (0, 2.5, 5, 10, 20 and 800 g a.e. ha^-1^)] in the first and second study, respectively. In the second study, two soil moistures (adequately-watered and water-stressed), and two *E*. *colona* biotypes, glyphosate-resistant and glyphosate-susceptible, were included. In both studies, plants that were treated with glyphosate at 2.5–40 g ha^-1^ grew taller and produced more leaves, tillers, inflorescences and seeds than the control treatment. In the first study, 5 g ha^-1^ glyphosate resulted in the maximum aboveground biomass (increase of 34% to 118%) compared with the control treatment. In the second study, the adequately-watered and glyphosate low dose treatments caused an increase in all the measured growth parameters for both biotypes. For example, total dry biomass was increased by 64% and 54% at 5 g ha^-1^ in the adequately-watered treatments for the resistant and susceptible biotypes, respectively, compared with the control treatment. All measured traits tended to decrease with increasing water stress and the stimulative growth of low doses of glyphosate could not compensate for the water stress effect. The results of both studies showed a hormetic effect of low doses of glyphosate on *E*. *colona* biotypes and such growth stimulation was significant in the range of 5 to 10 g ha^-1^ glyphosate. Water availability was found to be effective in modulating the stimulatory outcomes of glyphosate-induced hormesis. No significant difference was observed between the resistant and susceptible biotypes for hormesis phenomenon. The study showed the importance of precise herbicide application for suppressing weed growth and herbicide resistance evolution.

## Introduction

Herbicides are an important tool to combat weeds in contemporary agriculture. Both target and non-target plant species are frequently exposed to sub-lethal dose(s) of herbicides under field conditions. Sometimes, weeds receive low doses of herbicides for a number of reasons, such as their spatial distribution, adverse climatic during herbicide application (wind, rain, high temperature or moisture), incorrectly calibrated equipment and farmers’ mistakes [1]. Furthermore, non-target plants can be exposed to lower doses of herbicides via all of the above-mentioned reasons as well as from contact with treated weeds [2].

Hormesis refers to the stimulatory effect of low doses of an otherwise toxic substance [3, 4, 5]. It is a biphasic dose-response phenomenon to some pesticides and phytotoxins that are toxic/inhibitory at high doses and in contrast, may cause a beneficial stimulatory effect at low doses [3, 4, 6]. Hormetically boosted plants show a significant increase in growth due to enhanced biomass, shoot/plant length, photosynthesis, amino acids and protein content, and pest resistance [3, 7, 8, 9]. In recent times, several herbicides have been demonstrated to manifest hormetic effects on crops [8] and weeds [10, 11]. Nevertheless, such herbicide-induced hormesis is most extensively studied for glyphosate [12], the most commonly used herbicide worldwide [13]. Glyphosate-induced hormesis is of great concern because it can boost weed growth, reproductive output and competitive traits and thus can be one of the reasons for resistance development in weeds [12, 14, 15].

It is becoming increasingly apparent that besides its major commercial significance as a herbicide, glyphosate also has real and potential implications as a growth regulator at lower doses [2, 6, 16, 17]. Asman et al. (2003) postulated that glyphosate drift results in exposure of weeds growing at field edges to 1–10% (corresponding to 5–60 g ha^-1^) of recommended application rates under field conditions [1]. These doses can induce hormesis since they equate to the stimulatory dose range. Some greenhouse and field studies observed a yield increase of 12–175% in several plants when glyphosate was applied at 2–143 g ha^-1^ [2, 18]. Low glyphosate doses corresponding to 5–10% of field application rates stimulated barley seedling biomass by 25% [16]. These findings call for the need to develop practical approaches for real-time application in the field. Incorrect application, drift, rainfall following herbicide application, dew, low soil absorption, enhanced herbicide degradation in soil, and presence of waxy bloom or pubescence on leaves can dilute the herbicide [5]. Bott et al. (2011) [19] found that a bio-available dose of glyphosate in soil, after application of a toxic dose of glyphosate to the soil, was hermetic. In agro-ecosystems, hormetic responses to glyphosate by weeds, due to dilution of toxic doses of glyphosate by drift, rain, dew, or residues in the soil, can alter weed-crop competition in favor of either weeds or crops [11]. It is unequivocal that mechanisms and effects of hormesis will vary with herbicide, formulation, species and growth stage besides the interaction of biotic and abiotic factors affecting herbicide performance and species fitness. Thus, considerable research efforts are needed to gain a clear understanding of herbicide-induced hormetic effects in crops and their associated weeds. The magnitude of hormetic stimulation and its relevant parameters, underlying mechanisms and sub-toxic doses conducive to such effects need to be evaluated across a range of crops and agro-ecosystems.

The magnitude of hormetic response varies with herbicide, dose, formulation, environmental factors, plant species, growth stage and physiological status [9, 12, 20, 21]. Several environmental factors that cause changes to plant growth, such as temperature, light, CO_2_, nutrients and water availability, can affect the stimulatory outcomes of hormesis and its ecological and evolutionary implications [12, 15]. Studies pertaining to the influence of environmental factors on hormesis in weeds are rare; however, previous studies on barley (*Hordeum vulgare*) have shown that the hormetic effect of glyphosate was increased when light and CO_2_ concentration [22] or water supply [18]. were not limiting photosynthesis.

*Echinochloa colona* L. (Link) is a C_4_ annual summer grass that is native to Europe and India and can be either tetraploid or hexaploidy [23]. It is a serious weed of rice (*Oryza sativa* L.), sugarcane (*Saccharum* spp.), maize (*Zea mays* L.) and sorghum (*Sorghum bicolor* Moench.) crops. A single plant of this weed is capable of producing up to 42000 seeds, which allows effective dispersal of this weed. The first report of glyphosate resistance in *E*. *colona* in Australia was reported in a population from New South Wales in 2007 [24] and after that in 2009 in Queensland [25]. In Australia, *E*. *colona* is the second ranked weed in terms of the number of glyphosate-resistant populations after *Lolium rigidum* Gaud. [26]. Different glyphosate resistance mechanisms such as EPSPS target-site alteration (target site resistance) and reduced glyphosate uptake/translocation or enhanced vacuole sequestration (non target site resistance) have been detected in glyphosate-resistant weed species [27, 28, 29]. For glyphosate-resistant *E*. *colona*, three target-site EPSPS mutations have been documented [30, 31].

In an agricultural context, promotion of herbicide-resistant weeds via the hormetic effect of herbicides is of great concern [12, 14]. While previous studies revealed that intensive use of field rate doses of herbicides cannot directly select resistant weeds and evolve herbicide resistance, herbicides may have an indirect effect on promoting the growth of herbicide-resistant weeds via hormesis by low doses [17]. Other studies found that the hormetic response can be more evident in resistant weeds even at high herbicide doses [14, 32]. They observed that herbicide doses that are lethal to susceptible plants can cause a hormetic effect on resistant individuals [14, 32].

Although there is ever-growing consensus that the occurrence of weed resistance to glyphosate is increasing rapidly, information regarding the glyphosate-induced hormetic response of resistant weed biotypes is not readily available [33]. Limited information is available for hormetic effects of glyphosate in weeds, especially resistant biotypes and how this might influence overall weed management. We hypothesized that due to "selective hormesis", glyphosate-resistant and susceptible populations of *E*. *colona* may exhibit a differential hormetic response and that variations in water supply can modulate this response. The specific research questions of our studies were: (i) can low doses of glyphosate promote the growth traits and reproductive potential of *E*. *colona*? (ii) are the glyphosate-induced hormetic enhancements similar between glyphosate-resistant and susceptible populations of *E*. *colona*? (iii) is the dose that induces hormesis consistent for glyphosate-resistant and susceptible populations? and (iv) is hormesis affected by water stress?

## Materials and methods

### Seed description and seedling preparation

Seeds of *E*. *colona* biotypes for both studies were originally collected from the Gatton research field of the University of Queensland at Gatton, Queensland, Australia (approximately latitude 27.33° S, longitude 152.16° E and altitude 94 m a.s.l.) in 2015. The glyphosate-resistant biotype was confirmed to be resistant in a screenhouse study, in which plants were sprayed with different doses of glyphosate [34]. According to an experiment conducted by Mutii et al. (2019) at the Gatton research field, the glyphosate-resistant and susceptible biotypes were propagated by the cloning method that produces individual plants with the same genetic background as their parents [35]. The repeatability of the experiment is possible as the samples are identified and the populations are available for further studies.

### Study I

**Fig 1:**
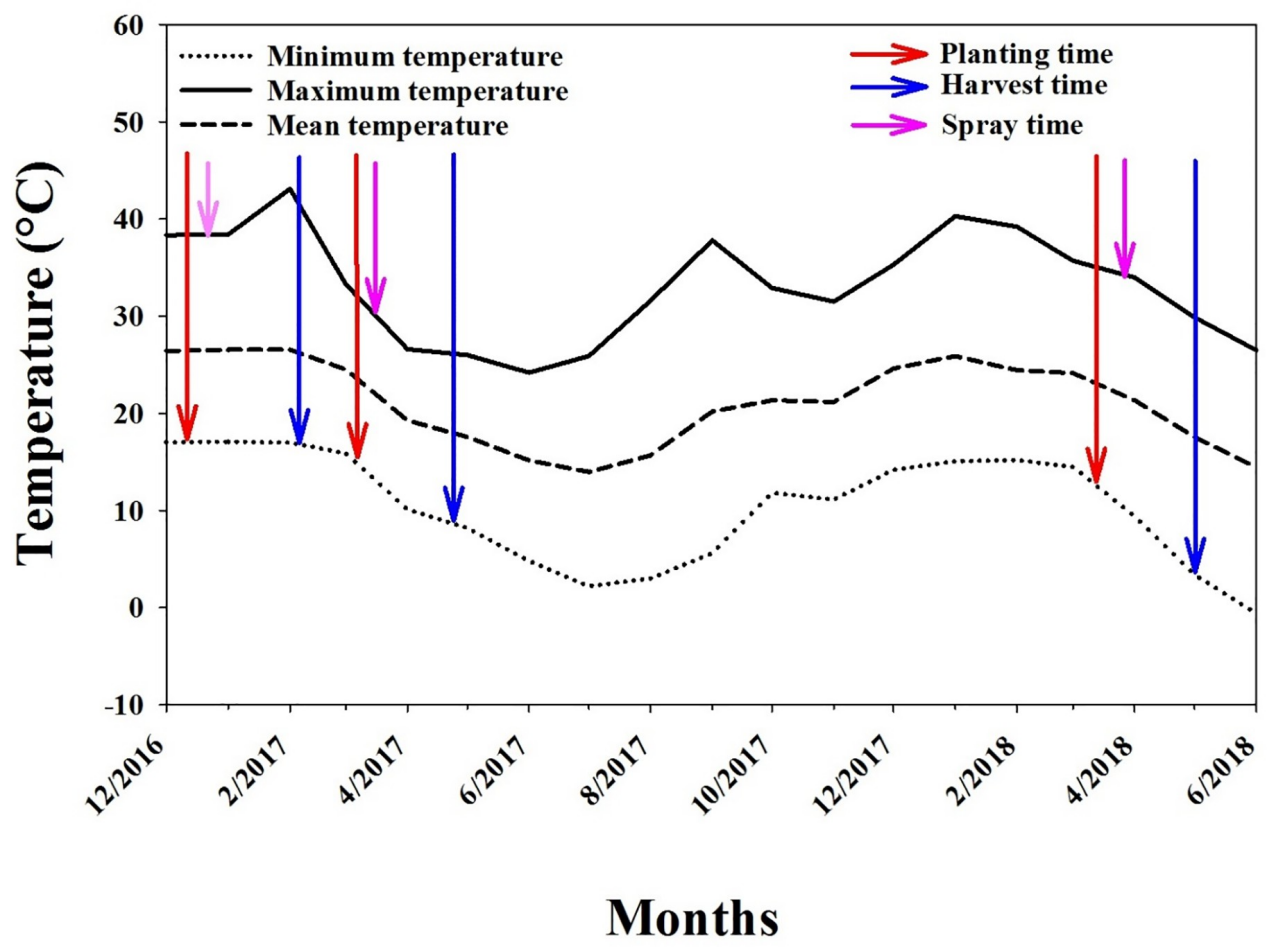
Mean, minimum and maximum temperatures during the studies conducted on the hormetic effect of glyphosate on *Echinochloa colona* in a naturally ventilated screenhouse at the Gatton Campus of the University of Queensland, Australia.

In 2016–2017, a pot study (with two trials) was conducted to evaluate the effect of different doses of glyphosate on *E*. *colona* in the screenhouse facility (a naturally ventilated shadehouse) at the Gatton Campus of the University of Queensland, Australia. In this study, a single population of *E*. *colona* was used, which was susceptible to glyphosate. Pots (20 cm diameter) were filled with potting mix and one two-leaf seedling of *E*. *colona* was transplanted in each pot. The mean, minimum and maximum temperatures during the course of these studies are presented in Fig 1. The pots were irrigated regularly and glyphosate was sprayed using a research track sprayer at different doses (0, 5, 10, 20, 40, 80 and 800 g a.e. ha^-1^) at the 3–4 leaf stage. A spray volume of 108 L ha^-1^ was used. Hormetic responses in terms of morphological growth characters such as plant height and number of tillers, leaves and inflorescences were measured at 2, 4, 6 and 8 weeks after spraying. Seed production and aboveground biomass were recorded at the end of the study.

### Study II

In 2018, a second pot study was conducted to assess the effect of different doses of glyphosate and soil moisture regimes on two *E*. *colona* biotypes (glyphosate-resistant and susceptible). Plants of both biotypes were grown in the screenhouse at two soil moisture conditions: adequately-watered (continuously sub-irrigated) and water-stressed (irrigated once a week). Plants were treated with different glyphosate doses (0, 2.5, 5, 10, 20 and 800 g a.e. ha^-1^) at the 3–4 leaf stage as outlined in Study I. Growth parameters such as plant height, and numbers of leaves, tillers and inflorescences were measured four times at a 10-day interval. At the end of the study, number of seeds and dry biomass were also measured. For dry biomass, plants were cut at the soil surface, placed in paper bags and dried in an oven at 70°C for 48 h.

### Statistical analyses

The first study was conducted using a completely randomized design with 10 replications. The second study was designed as factorial (biotypes × water regimes × glyphosate doses) based on a randomized completely block design with eight replications. The first study was done twice and each trial was analysed separately as significant differences were observed between the two runs. The second study was conducted once. Before analyses, the homogeneity and normality of data were checked and analysis of variance (ANOVA) was performed using SAS (version 9; SAS Institute Inc., Cary, NC, USA). Differences amongst treatment means were evaluated by Fisher’s protected Least Significant Differences (LSD, p≤0.05) test.

## Results

All measured growth characteristics were stimulated in the glyphosate low doses treatments.

### Study I

#### Height

In both trials, plants sprayed with 5, 10 and 20 g ha^-1^ glyphosate doses showed a significant (*p*<0.001) increase in the plant height compared with the control treatment (Table 1). No plants survived at 800 g ha^-1^ glyphosate in both trials. The maximum height was recorded at 10 g ha^-1^ glyphosate, that was 12% and 22% higher than the control treatment in the first and second trial, respectively.

**Table 1:** Effect of different glyphosate doses on height (cm) of *Echinocloa colona* plants in trials I and II (study I).

Dose (g a.e. ha^-1^)	Plant height (cm)	
	Trial Ι	Trial ΙΙ
**0**	69.3	57.3
**5**	76.1	70.0
**10**	77.9	70.1
**20**	73.6	65.2
**40**	72.2	63.2
**80**	75.0	55.3
**800**	-	-
**LSD (0.05)**	3.88	6.39

- did not survive.

#### Leaf number

In the first trial, the number of leaves per plant increased significantly (*p*<0.001) at 5 and 10 g ha^-1^ glyphosate (by 45% and 42%, respectively) compared with the control treatment (Table 2). However, in the second trial, this increase in the number of leaves was not significant. Generally, the number of produced leaves in the second trial was significantly lower than the first run.

**Table 2:** Effect of different glyphosate doses on leaf number of *Echinocloa colona* plants (number/plant) in trials I and II (study I).

Dose (g a.e. ha^-1^)	Leaf number per plant	
	Trial Ι	Trial ΙΙ
**0**	130.1	60.8
**5**	188.5	74.0
**10**	185.1	62.6
**20**	164.2	59.7
**40**	162.5	59.4
**80**	162.2	69.0
**800**	-	-
**LSD (0.05)**	47.92	14.48

- did not survive.

#### Tiller number

The highest number of tillers per plant in both trials was observed at 5 g ha^-1^ glyphosate treatment (Table 3). At this dose the tiller production increased significantly (*p*<0.001) by 59% and 47% compared with the control treatment in the first and second trial, respectively.

**Table 3:** Effect of different glyphosate doses on tillers number of *Echinocloa colona* plants (number/plant) in trials I and II (study I).

Dose (g a.e. ha^-1^)	Tiller number per plant	
	Trial Ι	Trial ΙΙ
**0**	66.4	47.9
**5**	105.3	70.5
**10**	92.9	59.1
**20**	87.2	54.5
**40**	86.6	53.9
**80**	75.1	60.4
**800**	-	-
**LSD (0.05)**	28.45	14.53

- did not survive.

#### Inflorescence and seed production

Compared with the control treatment, the number of inflorescences per plant was increased significantly (*p*<0.001) at 5 g ha^-1^ glyphosate treatment (by 64% and 45% in the first and second trials, respectively) (Table 4). Consequently, in the first and second trial, plants that were sprayed with 5 g ha^-1^ glyphosate produced 78% and 88% more seeds, respectively, than unsprayed (control) plants (Table 5).

**Table 4:** Effect of different glyphosate doses on inflorescence number of *Echinocloa colona* plants (number/plant) in trials I and II (study I).

Dose (g a.e. ha^-1^)	Inflorescence number per plant	
	Trial Ι	Trial ΙΙ
**0**	62.0	45.1
**5**	101.6	65.5
**10**	84.2	53.0
**20**	83.5	47.3
**40**	67.3	48.9
**80**	55.6	51.0
**800**	-	-
**LSD (0.05)**	27.16	13.07

- did not survive.

**Table 5:** Effect of different glyphosate doses on number of seeds per plant of *Echinocloa colona* in trials I and II (study I).

Dose (g a.e. ha^-1^)	Seed number per plant	
	Trial Ι	Trial ΙΙ
**0**	12439	4703
**5**	22183	8848
**10**	16257	7902
**20**	16238	6585
**40**	12678	5482
**80**	10884	4951
**800**	-	-
**LSD (0.05)**	7048.68	2291.05

- did not survive.

#### Biomass

*Echinochloa colona* biomass stimulation by low doses of glyphosate followed a similar trend in both trials with biomass significantly (*p*<0.001) increasing between 5 to 40 g ha^-1^ glyphosate compared with the control treatment (Table 6). In both trials, the greatest biomass was recorded for plants growing in pots that were sprayed with 5 g ha^-1^ glyphosate. This corresponds to a 34% and 118% increase in biomass in the first and second run, respectively, compared with the control treatment.

**Table 6:** Effect of different glyphosate doses on aboveground biomass of *Echinocloa colona* plants in trials I and II (study I).

Dose (g a.e. ha^-1^)	Above ground biomass (g per plant)	
	Trial Ι	Trial ΙΙ
**0**	33.4	10.0
**5**	44.9	21.8
**10**	40.2	19.8
**20**	38.6	17.3
**40**	39.6	14.6
**80**	30.4	11.0
**800**	-	-
**LSD (0.05)**	8.25	6.35

- did not survive.

### Study II

#### Height of glyphosate-resistant and susceptible biotypes

The interaction of biotypes × water regimes × glyphosate doses was significant (*p*<0.001) for plant height. Water availability and low doses of glyphosate affected the plant height of the glyphosate-resistant and susceptible biotypes (Table 7). Plants treated with 5 and 10 g ha^-1^ glyphosate in both water treatments were significantly taller than the control treatment for both biotypes. In the adequately-watered treatment, the tallest plants were observed at 5 g ha^-1^ glyphosate; however, in the water-stressed treatment, maximum height was observed at 20 g ha^-1^ glyphosate for both biotypes.

**Table 7:** Effect of different glyphosate doses and water levels on plant height (cm) of glyphosate-resistant and susceptible biotypes of *Echinochloa colona* (study II).

Dose (g a.e. ha^-1^)	Plant height (cm)			
	Susceptible		Resistant	
	Well water	Water stress	Well water	Water stress
**0**	47.4	38.8	42.4	36.2
**2.5**	49.0	39.7	45.2	38.2
**5**	56.0	43.5	55.3	40.5
**10**	53.8	45.8	51.2	40.2
**20**	50.7	48.8	48.5	42.4
**800**	-	-	24.2	21.8
**LSD (0.05)**	3.71			

#### Leaf number of glyphosate-resistant and susceptible biotypes

The interaction of biotypes × glyphosate doses and the interaction of water regimes × glyphosate dose were significant (*p*<0.001) for leaf number per plant. Despite a stimulation trend at all low doses of glyphosate, both glyphosate-resistant and susceptible biotypes of *E*. *colona* showed a significant increase in the number of leaves only at 5 g ha^-1^ glyphosate in the adequately-watered treatment compared with the control (Table 8). Leaf number was 30% and 27% greater in the resistant and susceptible biotypes, respectively, compared to their respective controls. In all treatments, adequately-watered plants significantly produced more leaves than the water-stressed plants of both biotypes. However, there was no significant difference for the number of leaves at 800 g ha^-1^ glyphosate between the adequately-watered and water-stressed treatments for the resistant biotype.

**Table 8:** Effect of different glyphosate doses and water levels on number of leaves per plant in the glyphosate-resistant and susceptible biotypes of *Echinochloa colona* (study II).

Dose (g a.e. ha^-1^)	Leaf number per plant			
	Susceptible		Resistant	
	Well water	Water stress	Well water	Water stress
**0**	96.8	56.7	91.8	54.8
**2.5**	95.7	62.3	99.0	60.0
**5**	123.2	80.6	119.5	70.8
**10**	117.5	85.7	113.7	70.0
**20**	109.3	86.3	87.3	77.0
**800**	-	-	26.5	25.5
**LSD (0.05)**	23.64			

#### Tiller number of glyphosate-resistant and susceptible biotypes

The interaction of biotypes × glyphosate doses and the interaction of water regimes × glyphosate dose were significant (*p*<0.001) for tiller number. The increase in the number of tillers at 5, 10 and 20 g ha^-1^ glyphosate was significant as compared to the control treatment in both moisture regimes and *E*. *colona* biotypes (Table 9). The highest number of tillers was recorded for plants of the susceptible biotype sprayed with 5 and 10 g ha^-1^ glyphosate and grown in the adequately-watered condition.

**Table 9:** Effect of different glyphosate doses and water levels on number of tillers per plant in the glyphosate-resistant and susceptible biotypes of *Echinochloa colona* (study II).

Dose (g a.e. ha-^1^)	Tiller number per plant			
	Susceptible		Resistant	
	Well water	Water stress	Well water	Water stress
**0**	24.16	20.16	19.66	14.16
**2.5**	26.16	21.5	21.33	16.16
**5**	32.5	23.66	29.66	21.16
**10**	32.66	25.66	28.33	22.33
**20**	28.66	26.33	29.83	24.16
**800**	-	-	3.83	2.83
**LSD (0.05)**	2.83			

#### Inflorescence and seed production of glyphosate-resistant and susceptible biotypes

The interaction of biotypes × water regimes × glyphosate doses was significant (*p*<0.001). All glyphosate low doses, except 2.5 g ha^-1^, caused a significant increase in the number of seeds produced per plant for both biotypes compared with the plants that were not sprayed (Tables 10 and 11). This increase was greater when the plants of both biotypes were grown under adequately adequately-watered conditions. Nevertheless, the inflorescence number produced by the susceptible biotype was higher than the resistant biotype at both water levels. At 5 g ha^-1^ glyphosate in the adequately adequately-watered condition, the number of inflorescences and seeds increased by 18% and 44%, respectively, for the glyphosate-susceptible biotype compared with the plants of the same biotype treated with 0 g ha^-1^ glyphosate. In the water-stressed condition the number of inflorescences and seeds increased by 14% and 22%, respectively (Tables 10 and 11). The glyphosate-resistant biotype showed a 16% and 64% increase in the number of inflorescences and seeds produced at the 5 g ha^-1^ glyphosate treatment under the adequately-watered condition, and 8% and 39% under the water-stressed condition. The maximum number of inflorescences and seeds for both biotypes in the adequately-watered and water-stressed conditions was observed at 5 and 20 g ha^-1^ glyphosate, respectively. The glyphosate-susceptible biotype produced more seeds than the resistant biotype at all treatments except 800 g ha^-1^ glyphosate. In the adequately-watered treatment, the glyphosate-susceptible biotype produced 23%, 8% and 34% more number of seeds per plant than the resistant biotype at 0, 5 and 10 g ha^-1^ glyphosate, respectively (Table 11).

**Table 10:** Effect of different glyphosate doses and water levels on inflorescence number of the glyphosate-resistant and susceptible biotypes of *Echinochloa colona* (study II).

Dose (g a.e. ha^-1^)	Inflorescence number per plant			
	Susceptible		Resistant	
	Well water	Water stress	Well water	Water stress
**0**	54.5	33.8	36.5	19.7
**2.5**	55.2	35.5	37.3	20.8
**5**	64.2	38.2	42.3	21.3
**10**	61.2	41.8	41.7	26.2
**20**	58.5	43.2	41.3	30.8
**800**	-	-	3.3	0.7
**LSD (0.05)**	2.83			

**Table 11:** Effect of different glyphosate doses and water levels on seed number of the glyphosate-resistant and susceptible biotypes of *Echinochloa colona* (study II).

Dose (g a.e. ha^-1^)	Seed number per plant			
	Susceptible		Resistant	
	Well water	Water stress	Well water	Water stress
**0**	14875	6729	12078	5197
**2.5**	15339	7632	12852	5288
**5**	21414	8213	19873	7236
**10**	21133	9239	15712	7769
**20**	17466	10658	14456	9121
**800**	-	-	140	64
**LSD (0.05)**	1287.89			

#### Biomass of glyphosate-resistant and susceptible biotypes

The interaction of water regimes × glyphosate doses was significant (*p*<0.001) for plant biomass. In the adequately-watered condition, at 5 g ha^-1^ glyphosate, the resistant and susceptible biotypes produced 64% and 54% more biomass than the control treatment, respectively. Maximum dry biomass for both biotypes was observed at 5 and 20 g ha^-1^ glyphosate in the adequately-watered and water-stressed conditions, respectively (Table 12). Water-stressed conditions caused a significant reduction in plant biomass at all glyphosate low doses. No significant difference was observed between the biomass of both biotypes except at the recommended glyphosate field dose (800 g ha^-1^) in both water conditions. Resistant plants that were sprayed with the recommended glyphosate field dose produced some biomass (2 g plant^-1^), whereas the susceptible plants did not survive. At 800 g ha^-1^ glyphosate, biomass accumulation by plants of the resistant biotype did not vary significantly between the adequately-watered and water-stressed treatments (Table 12).

**Table 12:** Effect of different glyphosate doses and water levels on biomass of the glyphosate-resistant and susceptible biotypes of *Echinochloa colona* (study II).

Dose (g a.e. ha^-1^)	Biomass (g plant ^-1^)			
	susceptible		Resistant	
	Well water	Water stress	Well water	Water stress
**0**	9.4	3.4	8.3	3.3
**2.5**	10.3	3.7	9.5	3.8
**5**	14.5	5.7	13.6	5.4
**10**	11.5	6.3	12.2	6.3
**20**	10.7	6.8	10.8	6.7
**800**	-	-	2.4	2.2
**LSD (0.05)**	2.04			

## Discussion

A trend of glyphosate-induced growth stimulation at low doses and growth inhibition at 800 g ha^-1^ was observed in both studies. Plants that were treated with glyphosate at a range of low doses (2.5–40 g ha^−1^) grew taller and produced more leaves, tillers, inflorescences and seeds in both studies. However, this stimulative growth was only significant between 5 to 10 g ha^-1^ glyphosate doses in both studies. The results showed that plants treated with low doses of glyphosate recorded greater biomass at 5 g ha^-1^ in the first study, and 5 and 10 g ha^-1^ in the second study for the adequately-watered and water-stressed treatments, respectively. The number of produced leaves, tillers, inflorescences and seeds per plant in the second trial of the first study was less than the first trial, presumably because of different temperatures during these runs.

Our results coincided with those of Schabenberger et al. (1999) [36], a dose-response study that analyzed the hormesis effect of different herbicides. They observed that 5 g ha^-1^ glyphosate led to a 30% increase in dry weight of *Echinochloa crus-galli* (L.) P. Beauv. This growth promotion following the application of low doses of glyphosate has been mentioned in previous studies [2, 3, 37, 38]. In addition to increased growth, previous literature mentions that applying glyphosate at low doses may lead to an accumulation of shikimic acid, increase in photosynthesis and stomatal opening, increased reproductive potential and reduced life cycle [22, 33]. Belz and Duke (2014) [12] postulated that low inhibition of EPSPS may enhance the elasticity of cell walls with a concurrent increase in cell elongation, owing to the preferential reduction in lignin biosynthesis.

Cedergreen and Olesen (2010) [22] reported that growth-induction by low glyphosate doses in barley (*Hordeum vulgare* L.) can be correlated with an increase in photosynthesis. Therefore, the positive effects on the photosynthetic process can be another possibility for explaining the hormetic effect of glyphosate. However, the physiological mechanisms of such increases in growth are not clear. Overcompensation that is mentioned in some hormesis studies is also an indirect mechanism of hormesis. The mechanism of overcompensation can effect on enzyme systems when plants exposure to minimal stress induced by herbicide(s) or a response to disturbed homeostasis. The overcompensation appears to promote the overall growth and health of plants, although this mechanism has not been proven for herbicides [12].

Water is a crucial factor that influences almost all physiological processes within a plant. Our results showed that all measured physiological traits have tended to decrease with water-deficiency and the glyphosate hormetic effect was more obvious in the adequately-watered treatment in both biotypes. The results showed that the maximum dry biomass for both biotypes was observed at 5 and 20 g ha^-1^ glyphosate in the adequately-watered and water-stressed treatments, respectively. Therefore, the hormesis effect of glyphosate at low doses can happen at higher doses in water deficit and it could be due to a lower translocation ability in the water-stressed condition.

Several environmental factors that can cause a change in plant growth, such as temperature, light, CO_2_, nutrients and water availability, can affect the stimulatory response of hormesis [12]. Water stress causes stomatal closure and decreased leaf water status, and consequently decreases stomatal conductance, CO_2_ assimilation and activity of photosynthetic enzymes [39]. Our results showed that water availability can modulate the stimulatory outcomes of glyphosate-induced hormesis in weeds. The highest magnitude of growth stimulation was realized at 5 and 10 g ha^-1^ glyphosate in the adequately-watered and water-stressed treatments, respectively. However, Cedergreen et al. (2009) [18] mentioned that glyphosate-induced growth via hormesis occurs only when drought is not a limiting factor for photosynthesis. Reduced soil moisture content resulted in a decrease in the efficacy of glyphosate [12, 40]. In our previous study, we observed that in the water stressed condition, glyphosate efficacy was decreased in both biotypes [34]. This response could be caused by less absorption and translocation of glyphosate as the herbicide is mainly translocated by vascular transportation [41]. The increased growth may also be attributed to better herbicide translocation under greater water supply. Our results showed that water availability can affect the plant biomass and growth parameters more than the hormetic effect of glyphosate at low doses.

In both weed biotypes, the adequately-watered and glyphosate low doses treatments caused an increase in all the measured growth parameters. Generally, in our study, both *E*. *colona* biotypes that were exposed to the low glyphosate doses showed approximately a similar stimulatory response and it was dose-dependent. Limited information about the glyphosate hormetic effects on glyphosate-resistant weeds is available. Brito et al. (2018) [33] mentioned that the critical dose range responsible for hormesis in herbicide-resistant plants is linked with the degree of susceptibility or resistance to the specific herbicide. A study on soybean observed that glyphosate-resistant soybean (transgenic) with an insensitive EPSPS enzyme did not exhibit a hormesis response to low doses of glyphosate, whereas non-resistant soybean (non-transgenic) with a sensitive EPSPS enzyme showed a significant stimulation [2]. In the same study they also discussed that the lack of a stimulatory effect of glyphosate in glyphosate-resistant soybean at any dose suggests a target-site resistance mechanism in the glyphosate-resistant soybean. In another study, Belz and Duke (2014) [12] reported that the dose responsible for hormetic effect in glyphosate-resistant weeds should be higher than in susceptible weeds, owing to a lower amount of herbicide that is able to reach the target site due to absorption and translocation differences resulting from the non-target site resistance mechanism.

Our observation is inconsistent with the above mentioned studies. The glyphosate-resistant biotype showed a significant growth increase at low glyphosate doses similar to the susceptible biotype and this could be supported by the findings of Han et al. (2016) [26] who identified EPSPS target-site mutations in two *E*. *colona* populations from the north-eastern region of Australia. These authors demonstrated that in relation to prevailing environmental conditions, these target-site mutations may show a different level of resistance in the polyploid weed species. In polyploid species, the likelihood of target-site mutation related herbicide resistance is lower than diploid species because of the dilution and/or expression effect by multiple EPSPS gene copies [26]. The hormesis response of the glyphosate-resistant biotype in our study could be due to a low level of resistance to glyphosate due to multiple copies of the EPSPS gene or the possibility of different herbicide resistance mechanisms. Although the mechanisms of its resistance relates to inhibition of the target enzyme (EPSPS), the presence of other EPSPS gene copies can respond to low doses of glyphosate to stimulate hormesis. Hormesis could theoretically cause better growth and increased competitiveness in both biotypes of *E*. *colona* without considering mechanical and precision application technologies for glyphosate spraying. There is a probability that growth stimulation via hormesis, makes weeds more competitive, more reproductive and more resistant to other control treatments and it can alter dynamics of weed-crop competition [10, 14, 42, 43]. If stimulated weeds are resistant to herbicides in the field, hormesis can directly facilitate the evolution of resistance by improving their growth and reproductive output [6,17, 42, 44].

Higher competitive traits of weeds stimulated by low doses of herbicides may cause changes in the weed population at the ecosystem scale. Therefore, considerable research efforts are needed to have a clear understanding of glyphosate and other herbicide-induced hormetic effects on crops and their associated weeds. In pot experiments, doses are in much more uniform conditions. However, doses received in fields are variable and unpredictable from one experiment to another. Little is known about the hormetic effect of herbicides on weeds under field conditions. It is important for field studies designed with many doses for different herbicides to estimate the hormetic effects in weeds of economic significance.

## Conclusions

Our results suggest that low doses of glyphosate can induce growth in both resistant and susceptible biotypes of *E*. *colona* and this is pronounced in adequately-watered conditions. The hormesis effect that occurs at low doses of glyphosate can also happen at higher doses in water deficit conditions. Both *E*. *colona* biotypes showed an approximately similar stimulatory response to the low glyphosate doses; however, the physiological and biochemical mechanisms behind the glyphosate-hormetic effects are not determined. These findings show the importance of diversity in weed control practices (not only chemical control) because a sole reliance on glyphosate may exert a strong selection pressure and result in the evolution of resistant biotypes.

## Supporting information

S1 Data (XLSX)

S2 Data (XLSX)

S1 Table ANOVA on height of *Echinocloa colona* plants in data study Ι. (DOCX)

S2 Table ANOVA on height of *Echinocloa colona* plants in data study Ι trial Ι. (DOCX)

S3 Table ANOVA on height of *Echinocloa colona* plants in data study Ι trial Ι. (DOCX)

S4 Table ANOVA on number of leaves of *Echinocloa colona* plants data in study Ι. (DOCX)

S5 Table ANOVA on number of leaves of *Echinocloa colona* plants data in study Ι trial Ι. (DOCX)

S6 Table ANOVA on number of leaves of *Echinocloa colona* plants data in study Ι trial ΙΙ. (DOCX)

S7 Table ANOVA on number of tillers of *Echinocloa colona* plants data in study Ι. (DOCX)

S8 Table ANOVA on number of tillers of *Echinocloa colona* plants data in study Ι trial Ι. (DOCX)

S9 Table ANOVA on number of tillers of *Echinocloa colona* plants data in study Ι trial ΙΙ. (DOCX)

S10 Table ANOVA on number of inflorescences of *Echinocloa colona* plants data in study Ι. (DOCX)

S11 Table ANOVA on number of inflorescences of *Echinocloa colona* plants data in study Ι trial Ι. (DOCX)

S12 Table ANOVA on number of inflorescences of *Echinocloa colona* plants data in study Ι trial ΙΙ. (DOCX)

S13 Table ANOVA on biomass of *Echinocloa colona* plants data in study Ι. (DOCX)

S14 Table ANOVA on biomass of *Echinocloa colona* plants data in study Ι trial Ι. (DOCX)

S15 Table ANOVA on biomass of *Echinocloa colona* plants data in study Ι trial ΙΙ. (DOCX)

S16 Table ANOVA on seed production of *Echinocloa colona* plants data in study Ι. (DOCX)

S17 Table ANOVA on seed production of *Echinocloa colona* plants data in study Ι trial Ι. (DOCX)

S18 Table ANOVA on seed production of *Echinocloa colona* plants data in study Ι trial ΙΙ. (DOCX)

S19 Table ANOVA on glyphosate doses and water levels on plant height of glyphosate-resistant and susceptible biotypes of *Echinochloa colona* data in study ΙΙ. (DOCX)

S20 Table ANOVA on glyphosate doses and water levels on number of leaves per plant in the glyphosate-resistant and susceptible biotypes of *Echinochloa colona* data in study ΙΙ. (DOCX)

S21 Table ANOVA on glyphosate doses and water levels on number of tillers per plant in the glyphosate-resistant and susceptible biotypes of *Echinochloa colona* data in study ΙΙ. (DOCX)

S22 Table ANOVA on glyphosate doses and water levels on number of inflorescence per plant in the glyphosate-resistant and susceptible biotypes of *Echinochloa colona* data in study ΙΙ. (DOCX)

S23 Table ANOVA on glyphosate doses and water levels on number of seed per plant in the glyphosate-resistant and susceptible biotypes of *Echinochloa colona* data in study ΙΙ. (DOCX)

S24 Table ANOVA on glyphosate doses and water levels on plant biomass of glyphosate-resistant and susceptible biotypes of *Echinochloa colona* data in study ΙΙ. (DOCX)
