# Supplementary material for: Response of glyphosate-resistant and susceptible biotypes of Echinochloa colona to low doses of glyphosate in different soil moisture conditions
Source: PLoS One. 2020 May 20;15(5):e0233428. doi: 10.1371/journal.pone.0233428 (PMC7239466; doi:10.1371/journal.pone.0233428)
Supplement: S1 Table — (DOCX) [file pone.0233428.s003.docx]

| Table 1. ANOVA on height of *Echinocloa colona* plants in data study Ι | | | | | | | | | | |
| --- | --- | --- | --- | --- | --- | --- | --- | --- | --- | --- |
| **EFFECT** | **SS** | **DF** | **MS** | **F** | **ProbF** | **Sign.** | **S.E.M.** | **S.E.D.** | **L.S.D. (0.05)** | **L.S.D. (0.01)** |
| Replications | 76803.09524 | 9 | 8533.677249 | 0.553641 | 0.910248 |  |  |  |  |  |
| Treatments | 8296128.452 | 6 | 1382688.075 | 413.7594 | 2.71E-76 | ** | 12.92627 | 18.2805 | 36.20358 | 47.86758 |
| runs | 283200.0794 | 1 | 283200.0794 | 84.74557 | 1.62E-15 | ** | 6.909381 | 9.771341 | 19.35163 | 25.5863 |
| Treatments x Runs | 106966.3095 | 6 | 17827.71825 | 5.334816 | 6.74E-05 | ** | 18.2805 | 25.85254 | 51.1996 | 67.69498 |
| Residual | 390986.9048 | 117 | 3341.768417 |  |  |  |  |  |  |  |
| Total | 9154084.841 | 139 | 65856.72548 |  |  |  |  |  |  |  |
| C.V. (%) = 9.80232262351826 | |  |  |  |  |  |  |  |  |  |
