# Supplementary material for: Response of glyphosate-resistant and susceptible biotypes of Echinochloa colona to low doses of glyphosate in different soil moisture conditions
Source: PLoS One. 2020 May 20;15(5):e0233428. doi: 10.1371/journal.pone.0233428 (PMC7239466; doi:10.1371/journal.pone.0233428)
Supplement: S2 Table — (DOCX) [file pone.0233428.s004.docx]

| Table 2. ANOVA on height of *Echinocloa colona* plants in data study Ι trial Ι | | | | | |
| --- | --- | --- | --- | --- | --- |
| **EFFECT** | **SS** | **DF** | **MS** | **F** | **ProbF** |
| Replications | 131.85 | 9 | 14.65 | 0.788058096 |  |
| Treatments | 461.95 | 5 | 92.39 | 4.969876278 | 0.001045354** |
| Residual | 836.55 | 45 | 18.59 |  |  |
| Total | 1430.35 | 59 | 24.24322034 |  |  |
| C.V. (%): 5.8225688419474 | |  |  |  |  |
| S.E.M.: 1.36345150262121 | |  |  |  |  |
| S.E.D.: 1.92821160664489 | |  |  |  |  |
| LSD (p<0.05): 3.88361753142284 | | |  |  |  |
| LSD (p<0.01): 5.1860890514164 | | |  |  |  |
