# Supplementary material for: Response of glyphosate-resistant and susceptible biotypes of Echinochloa colona to low doses of glyphosate in different soil moisture conditions
Source: PLoS One. 2020 May 20;15(5):e0233428. doi: 10.1371/journal.pone.0233428 (PMC7239466; doi:10.1371/journal.pone.0233428)
Supplement: S3 Table — (DOCX) [file pone.0233428.s005.docx]

| Table 3. ANOVA on height of *Echinocloa colona* plants in data study Ι trial ΙΙ | | | | | |
| --- | --- | --- | --- | --- | --- |
| **EFFECT** | **SS** | **DF** | **MS** | **F** | **ProbF** |
| Replications | 1441.052344 | 9 | 160.1169271 | 3.175506613 |  |
| Treatments | 1932.792969 | 5 | 386.5585938 | 7.666393512 | 2.78634E-05** |
| Residual | 2269.011719 | 45 | 50.42248264 |  |  |
| Total | 5642.857031 | 59 | 95.6416446 |  |  |
| C.V. (%): 11.1703926924232 | |  |  |  |  |
| S.E.M.: 2.24549510440101 | |  |  |  |  |
| S.E.D.: 3.1756096308863 | |  |  |  |  |
| LSD (p<0.05): 6.39600611933074 | |  |  |  |  |
| LSD (p<0.01): 8.54107209061363 | |  |  |  |  |
