# Supplementary material for: Response of glyphosate-resistant and susceptible biotypes of Echinochloa colona to low doses of glyphosate in different soil moisture conditions
Source: PLoS One. 2020 May 20;15(5):e0233428. doi: 10.1371/journal.pone.0233428 (PMC7239466; doi:10.1371/journal.pone.0233428)
Supplement: S4 Table — (DOCX) [file pone.0233428.s006.docx]

| Table 4. ANOVA on number of leaves of *Echinocloa colona* plants data in study Ι | | | | | | | | | | |
| --- | --- | --- | --- | --- | --- | --- | --- | --- | --- | --- |
| **EFFECT** | **SS** | **DF** | **MS** | **F** | **ProbF** | **Sign.** | **S.E.M.** | **S.E.D.** | **L.S.D. (0.05)** | **L.S.D. (0.01)** |
| Replications | 6203.635714 | 9 | 689.2928571 | 0.546958 | 0.837389 |  |  |  |  |  |
| Treatments | 241100.4714 | 6 | 40183.4119 | 31.88579 | 1.71E-22 | ** | 7.937977 | 11.22599 | 22.23249 | 29.39531 |
| runs | 263264.5786 | 1 | 263264.5786 | 208.9021 | 8.6E-28 | ** | 4.243027 | 6.000547 | 11.88377 | 15.71246 |
| Treatments x Runs | 52510.87143 | 6 | 8751.811905 | 6.944617 | 2.48E-06 | ** | 11.22599 | 15.87595 | 31.4415 | 41.57125 |
| Residual | 147446.8643 | 117 | 1260.229609 |  |  |  |  |  |  |  |
| Total | 710526.4214 | 139 | 5111.700874 |  |  |  |  |  |  |  |
| C.V. (%) = 36.0638545300273 | |  |  |  |  |  |  |  |  |  |
