# Supplementary material for: Response of glyphosate-resistant and susceptible biotypes of Echinochloa colona to low doses of glyphosate in different soil moisture conditions
Source: PLoS One. 2020 May 20;15(5):e0233428. doi: 10.1371/journal.pone.0233428 (PMC7239466; doi:10.1371/journal.pone.0233428)
Supplement: S5 Table — (DOCX) [file pone.0233428.s007.docx]

| Table 5. ANOVA on number of leaves of *Echinocloa colona* plants data in study Ι trial Ι | | | | | |
| --- | --- | --- | --- | --- | --- |
| **EFFECT** | **SS** | **DF** | **MS** | **F** | **ProbF** |
| Replications | 8173.733333 | 9 | 908.1925926 | 0.320794527 |  |
| Treatments | 21878.73333 | 5 | 4375.746667 | 1.545614435 | 0.194982282** |
| Residual | 127398.2667 | 45 | 2831.072593 |  |  |
| Total | 157450.7333 | 59 | 2668.656497 |  |  |
| C.V. (%): 32.162698696117 | |  |  |  |  |
| S.E.M.: 16.8257914898307 | |  |  |  |  |
| S.E.D.: 23.7952625225804 | |  |  |  |  |
| LSD (p<0.05): 47.9261188860385 | |  |  |  |  |
| LSD (p<0.01): 63.999381612819 | |  |  |  |  |
