# Supplementary material for: Response of glyphosate-resistant and susceptible biotypes of Echinochloa colona to low doses of glyphosate in different soil moisture conditions
Source: PLoS One. 2020 May 20;15(5):e0233428. doi: 10.1371/journal.pone.0233428 (PMC7239466; doi:10.1371/journal.pone.0233428)
Supplement: S6 Table — (DOCX) [file pone.0233428.s008.docx]

| Table 6. ANOVA on number of leaves of *Echinocloa colona* plants data in study Ι trial ΙΙ | | | | | |
| --- | --- | --- | --- | --- | --- |
| **EFFECT** | **SS** | **DF** | **MS** | **F** | **ProbF** |
| Replications | 6485.0415 | 9 | 720.5601667 | 2.786554712 |  |
| Treatments | 1703.1675 | 5 | 340.6335 | 1.317299968 | 0.273913602* |
| Residual | 11636.3075 | 45 | 258.5846111 |  |  |
| Total | 19824.5165 | 59 | 336.0087542 |  |  |
| C.V. (%): 25.0729964230489 | |  |  |  |  |
| S.E.M.: 5.08512154339609 | |  |  |  |  |
| S.E.D.: 7.19144785298636 | |  |  |  |  |
| LSD (p<0.05): 14.4843194916597 | |  |  |  |  |
| LSD (p<0.01): 19.342010413006 | |  |  |  |  |
