# Supplementary material for: Response of glyphosate-resistant and susceptible biotypes of Echinochloa colona to low doses of glyphosate in different soil moisture conditions
Source: PLoS One. 2020 May 20;15(5):e0233428. doi: 10.1371/journal.pone.0233428 (PMC7239466; doi:10.1371/journal.pone.0233428)
Supplement: S7 Table — (DOCX) [file pone.0233428.s009.docx]

| Table 7. ANOVA on number of tillers of *Echinocloa colona* plants data in study Ι | | | | | | | | | | |
| --- | --- | --- | --- | --- | --- | --- | --- | --- | --- | --- |
| **EFFECT** | **SS** | **DF** | **MS** | **F** | **ProbF** | **Sign.** | **S.E.M.** | **S.E.D.** | **L.S.D. (0.05)** | **L.S.D. (0.01)** |
| Replications | 2793.774603 | 9 | 310.4194004 | 0.571536 | 0.818085 |  |  |  |  |  |
| Treatments | 98233.21164 | 6 | 16372.20194 | 30.14406 | 1.23E-21 | ** | 5.2112 | 7.369749 | 14.5954 | 19.29772 |
| runs | 19957.84268 | 1 | 19957.84268 | 36.74584 | 1.69E-08 | ** | 2.785503 | 3.939297 | 7.801571 | 10.31506 |
| Treatments x Runs | 5287.633862 | 6 | 881.2723104 | 1.622575 | 0.146888 |  | 7.369749 | 10.4224 | 20.64102 | 27.2911 |
| Residual | 63546.44762 | 117 | 543.1320309 |  |  |  |  |  |  |  |
| Total | 189818.9104 | 139 | 1365.603672 |  |  |  |  |  |  |  |
| C.V. (%) = 37.94555017029 | |  |  |  |  |  |  |  |  |  |
