# Supplementary material for: Response of glyphosate-resistant and susceptible biotypes of Echinochloa colona to low doses of glyphosate in different soil moisture conditions
Source: PLoS One. 2020 May 20;15(5):e0233428. doi: 10.1371/journal.pone.0233428 (PMC7239466; doi:10.1371/journal.pone.0233428)
Supplement: S8 Table — (DOCX) [file pone.0233428.s010.docx]

| Table 8. ANOVA on number of tillers of *Echinocloa colona* plants data in study Ι trial Ι | | | | | |
| --- | --- | --- | --- | --- | --- |
| **EFFECT** | **SS** | **DF** | **MS** | **F** | **ProbF** |
| Replications | 3922.446296 | 9 | 435.8273663 | 1.67290244 |  |
| Treatments | 2940.484979 | 5 | 588.0969959 | 2.257382109 | 0.06475634* |
| Residual | 11723.47593 | 45 | 260.5216872 |  |  |
| Total | 18586.4072 | 59 | 315.0238509 |  |  |
| C.V. (%): 27.9617897448271 | |  |  |  |  |
| S.E.M.: 5.10413251437301 | |  |  |  |  |
| S.E.D.: 7.2183334259758 | |  |  |  |  |
| LSD (p<0.05): 14.5384698153298 | |  |  |  |  |
| LSD (p<0.01): 19.4143214473558 | |  |  |  |  |
