# Supplementary material for: Response of glyphosate-resistant and susceptible biotypes of Echinochloa colona to low doses of glyphosate in different soil moisture conditions
Source: PLoS One. 2020 May 20;15(5):e0233428. doi: 10.1371/journal.pone.0233428 (PMC7239466; doi:10.1371/journal.pone.0233428)
Supplement: S9 Table — (DOCX) [file pone.0233428.s011.docx]

| Table 9. ANOVA on number of tillers of *Echinocloa colona* plants data in study Ι trial ΙΙ | | | | | |
| --- | --- | --- | --- | --- | --- |
| **EFFECT** | **SS** | **DF** | **MS** | **F** | **ProbF** |
| Replications | 5796.416667 | 9 | 644.0462963 | 0.645511128 |  |
| Treatments | 9238.283333 | 5 | 1847.656667 | 1.851859015 | 0.121962545** |
| Residual | 44897.88333 | 45 | 997.7307407 |  |  |
| Total | 59932.58333 | 59 | 1015.806497 |  |  |
| C.V. (%): 36.9077422596891 | |  |  |  |  |
| S.E.M.: 9.98864725946783 | |  |  |  |  |
| S.E.D.: 14.1260804241003 | |  |  |  |  |
| LSD (p<0.05): 28.4513864537837 | | |  |  |  |
| LSD (p<0.01): 37.9932942911414 | | |  |  |  |
