# Supplementary material for: Response of glyphosate-resistant and susceptible biotypes of Echinochloa colona to low doses of glyphosate in different soil moisture conditions
Source: PLoS One. 2020 May 20;15(5):e0233428. doi: 10.1371/journal.pone.0233428 (PMC7239466; doi:10.1371/journal.pone.0233428)
Supplement: S10 Table — (DOCX) [file pone.0233428.s012.docx]

| Table 10. ANOVA on number of inflorescences of *Echinocloa colona* plants data in study Ι | | | | | | | | | | |
| --- | --- | --- | --- | --- | --- | --- | --- | --- | --- | --- |
| **EFFECT** | **SS** | **DF** | **MS** | **F** | **ProbF** | **Sign.** | **S.E.M.** | **S.E.D.** | **L.S.D. (0.05)** | **L.S.D. (0.01)** |
| Replications | 4611.142857 | 9 | 512.3492063 | 0.963461 | 0.473861 |  |  |  |  |  |
| Treatments | 82938.64286 | 6 | 13823.10714 | 25.99403 | 1.81E-19 | ** | 5.156452 | 7.292325 | 14.44206667 | 19.09498189 |
| runs | 14688.25714 | 1 | 14688.25714 | 27.62093 | 6.72E-07 | ** | 2.75624 | 3.897911 | 7.71960935 | 10.20669715 |
| Treatments x Runs | 6473.842857 | 6 | 1078.97381 | 2.028985 | 0.067143 |  | 7.292325 | 10.3129 | 20.42416656 | 27.00438236 |
| Residual | 62218.25714 | 117 | 531.7799756 |  |  |  |  |  |  |  |
| Total | 170930.1429 | 139 | 1229.713258 |  |  |  |  |  |  |  |
| C.V. (%) = 42.2019569759896 | |  |  |  |  |  |  |  |  |  |
