# Supplementary material for: Response of glyphosate-resistant and susceptible biotypes of Echinochloa colona to low doses of glyphosate in different soil moisture conditions
Source: PLoS One. 2020 May 20;15(5):e0233428. doi: 10.1371/journal.pone.0233428 (PMC7239466; doi:10.1371/journal.pone.0233428)
Supplement: S11 Table — (DOCX) [file pone.0233428.s013.docx]

| Table 11. ANOVA on number of inflorescences of *Echinocloa colona* plants data in study Ι trial Ι | | | | | |
| --- | --- | --- | --- | --- | --- |
| **EFFECT** | **SS** | **DF** | **MS** | **F** | **ProbF** |
| Replications | 5886.266667 | 9 | 654.0296296 | 3.103218006 |  |
| Treatments | 2633.2 | 5 | 526.64 | 2.498783934 | 0.044324919* |
| Residual | 9484.133333 | 45 | 210.7585185 |  |  |
| Total | 18003.6 | 59 | 305.1457627 |  |  |
| C.V. (%): 28.0261091338916 | |  |  |  |  |
| S.E.M.: 4.5908443506453 | |  |  |  |  |
| S.E.D.: 6.49243434342648 | |  |  |  |  |
| LSD (p<0.05): 13.0764340131817 | |  |  |  |  |
| LSD (p<0.01): 17.4619541493533 | |  |  |  |  |
