# Supplementary material for: Response of glyphosate-resistant and susceptible biotypes of Echinochloa colona to low doses of glyphosate in different soil moisture conditions
Source: PLoS One. 2020 May 20;15(5):e0233428. doi: 10.1371/journal.pone.0233428 (PMC7239466; doi:10.1371/journal.pone.0233428)
Supplement: S12 Table — (DOCX) [file pone.0233428.s014.docx]

| Table 12. ANOVA on number of inflorescences of *Echinocloa colona* plants data in study Ι trial ΙΙ | | | | | |
| --- | --- | --- | --- | --- | --- |
| **EFFECT** | **SS** | **DF** | **MS** | **F** | **ProbF** |
| Replications | 10529.26667 | 9 | 1169.918519 | 1.286261332 |  |
| Treatments | 14661.6 | 5 | 2932.32 | 3.223925231 | 0.014273825** |
| Residual | 40929.73333 | 45 | 909.5496296 |  |  |
| Total | 66120.6 | 59 | 1120.688136 |  |  |
| C.V. (%): 39.8398157436722 | |  |  |  |  |
| S.E.M.: 9.53703113987593 | |  |  |  |  |
| S.E.D.: 13.4873987827871 | |  |  |  |  |
| LSD (p<0.05): 27.1650155955988 | |  |  |  |  |
| LSD (p<0.01): 36.2755057165159 | |  |  |  |  |
