# Supplementary material for: Response of glyphosate-resistant and susceptible biotypes of Echinochloa colona to low doses of glyphosate in different soil moisture conditions
Source: PLoS One. 2020 May 20;15(5):e0233428. doi: 10.1371/journal.pone.0233428 (PMC7239466; doi:10.1371/journal.pone.0233428)
Supplement: S13 Table — (DOCX) [file pone.0233428.s015.docx]

| Table 13. ANOVA on biomass of *Echinocloa colona* plants data in study Ι | | | | | | | | | | |
| --- | --- | --- | --- | --- | --- | --- | --- | --- | --- | --- |
| **EFFECT** | **SS** | **DF** | **MS** | **F** | **ProbF** | **Sign.** | **S.E.M.** | **S.E.D.** | **L.S.D. (0.05)** | **L.S.D. (0.01)** |
| Replications | 649.4885206 | 9 | 72.16539118 | 1.176267 | 0.316566 |  |  |  |  |  |
| Treatments | 14666.71583 | 6 | 2444.452638 | 39.8436 | 4.38E-26 | ** | 1.751445 | 2.476917 | 4.905405 | 6.485819 |
| runs | 12548.59268 | 1 | 12548.59268 | 204.537 | 1.9E-27 | ** | 0.936187 | 1.323968 | 2.622049 | 3.466816 |
| Treatments x Runs | 2203.208051 | 6 | 367.2013419 | 5.985235 | 1.75E-05 | ** | 2.476917 | 3.50289 | 6.937291 | 9.172333 |
| Residual | 7178.090542 | 117 | 61.35120121 |  |  |  |  |  |  |  |
| Total | 37246.09562 | 139 | 267.9575224 |  |  |  |  |  |  |  |
| C.V. (%) = 34.0969889228064 | |  |  |  |  |  |  |  |  |  |
