# Supplementary material for: Response of glyphosate-resistant and susceptible biotypes of Echinochloa colona to low doses of glyphosate in different soil moisture conditions
Source: PLoS One. 2020 May 20;15(5):e0233428. doi: 10.1371/journal.pone.0233428 (PMC7239466; doi:10.1371/journal.pone.0233428)
Supplement: S14 Table — (DOCX) [file pone.0233428.s016.docx]

| Table 14. ANOVA on biomass of *Echinocloa colona* plants data in study Ι trial Ι | | | | | |
| --- | --- | --- | --- | --- | --- |
| **EFFECT** | **SS** | **DF** | **MS** | **F** | **ProbF** |
| Replications | 990.6691417 | 9 | 110.0743491 | 1.31196667 |  |
| Treatments | 1356.469668 | 5 | 271.2939337 | 3.23352899 | 0.014063011** |
| Residual | 3775.511848 | 45 | 83.9002633 |  |  |
| Total | 6122.650658 | 59 | 103.77374 |  |  |
| C.V. (%): 24.2026872810673 |  |  |  |  |  |
| S.E.M.: 2.89655421658732 |  |  |  |  |  |
| S.E.D.: 4.09634625724676 |  |  |  |  |  |
| LSD (p<0.05): 8.25046487875007 | |  |  |  |  |
| LSD (p<0.01): 11.0174715276623 | |  |  |  |  |
