# Supplementary material for: Response of glyphosate-resistant and susceptible biotypes of Echinochloa colona to low doses of glyphosate in different soil moisture conditions
Source: PLoS One. 2020 May 20;15(5):e0233428. doi: 10.1371/journal.pone.0233428 (PMC7239466; doi:10.1371/journal.pone.0233428)
Supplement: S15 Table — (DOCX) [file pone.0233428.s017.docx]

| Table 15. ANOVA on biomass of *Echinocloa colona* plants data in study Ι trial ΙΙ | | | | | |
| --- | --- | --- | --- | --- | --- |
| **EFFECT** | **SS** | **DF** | **MS** | **F** | **ProbF** |
| Replications | 824.5939483 | 9 | 91.62154981 | 1.843241286 |  |
| Treatments | 1108.869208 | 5 | 221.7738417 | 4.461643596 | 0.002191323** |
| Residual | 2236.804142 | 45 | 49.7067587 |  |  |
| Total | 4170.267298 | 59 | 70.68249658 |  |  |
| C.V. (%): 44.7491427949817 |  |  |  |  |  |
| S.E.M.: 2.22950126045498 |  |  |  |  |  |
| S.E.D.: 3.15299091986334 |  |  |  |  |  |
| LSD (p<0.05): 6.35044969680729 | |  |  |  |  |
| LSD (p<0.01): 8.48023714428872 | |  |  |  |  |
