# Supplementary material for: Response of glyphosate-resistant and susceptible biotypes of Echinochloa colona to low doses of glyphosate in different soil moisture conditions
Source: PLoS One. 2020 May 20;15(5):e0233428. doi: 10.1371/journal.pone.0233428 (PMC7239466; doi:10.1371/journal.pone.0233428)
Supplement: S16 Table — (DOCX) [file pone.0233428.s018.docx]

| Table 16. ANOVA on seed production of *Echinocloa colona* plants data in study Ι | | | | | | | | | | |
| --- | --- | --- | --- | --- | --- | --- | --- | --- | --- | --- |
| **EFFECT** | **SS** | **DF** | **MS** | **F** | **ProbF** | **Sign.** | **S.E.M.** | **S.E.D.** | **L.S.D. (0.05)** | **L.S.D. (0.01)** |
| Replications | 182702792.6 | 9 | 20300310.29 | 0.654848 | 0.747899 |  |  |  |  |  |
| Treatments | 2795184208 | 6 | 465864034.7 | 15.02785 | 1.09E-12 | ** | 1244.991 | 1760.683 | 3486.94 | 4610.356 |
| runs | 1946890440 | 1 | 1946890440 | 62.80283 | 1.49E-12 | ** | 665.4756 | 941.1246 | 1863.848 | 2464.339 |
| Treatments x Runs | 491259823.6 | 6 | 81876637.26 | 2.641178 | 0.019405 | * | 1760.683 | 2489.982 | 4931.278 | 6520.027 |
| Residual | 3627005162 | 117 | 31000044.12 |  |  |  |  |  |  |  |
| Total | 9043042426 | 139 | 65057859.18 |  |  |  |  |  |  |  |
| C.V. (%) = 60.3551862926543 | |  |  |  |  |  |  |  |  |  |
