# Supplementary material for: Response of glyphosate-resistant and susceptible biotypes of Echinochloa colona to low doses of glyphosate in different soil moisture conditions
Source: PLoS One. 2020 May 20;15(5):e0233428. doi: 10.1371/journal.pone.0233428 (PMC7239466; doi:10.1371/journal.pone.0233428)
Supplement: S17 Table — (DOCX) [file pone.0233428.s019.docx]

| Table 17. ANOVA on seed production of *Echinocloa colona* plants data in study Ι trial Ι | | | | | |
| --- | --- | --- | --- | --- | --- |
| **EFFECT** | **SS** | **DF** | **MS** | **F** | **ProbF** |
| Replications | 567077648 | 9 | 63008627.56 | 1.028908544 |  |
| Treatments | 835212660.6 | 5 | 167042532.1 | 2.727745312 | 0.030945238** |
| Residual | 2755724265 | 45 | 61238317 |  |  |
| Total | 4158014574 | 59 | 70474823.28 |  |  |
| C.V. (%): 51.7793597201789 |  |  |  |  |  |
| S.E.M.: 2474.63769065675 |  |  |  |  |  |
| S.E.D.: 3499.66618408642 |  |  |  |  |  |
| LSD (p<0.05): 7048.68952132015 | |  |  |  |  |
| LSD (p<0.01): 9412.64974153085 | |  |  |  |  |
