# Supplementary material for: Response of glyphosate-resistant and susceptible biotypes of Echinochloa colona to low doses of glyphosate in different soil moisture conditions
Source: PLoS One. 2020 May 20;15(5):e0233428. doi: 10.1371/journal.pone.0233428 (PMC7239466; doi:10.1371/journal.pone.0233428)
Supplement: S18 Table — (DOCX) [file pone.0233428.s020.docx]

| Table 18. ANOVA on seed production of *Echinocloa colona* plants data in study Ι trial ΙΙ | | | | | |
| --- | --- | --- | --- | --- | --- |
| **EFFECT** | **SS** | **DF** | **MS** | **F** | **ProbF** |
| Replications | 195773430.8 | 9 | 21752603.43 | 3.362272441 |  |
| Treatments | 141066676.6 | 5 | 28213335.33 | 4.360899611 | 0.002543039** |
| Residual | 291132610.9 | 45 | 6469613.576 |  |  |
| Total | 627972718.4 | 59 | 10643605.4 |  |  |
| C.V. (%): 39.6693499831467 |  |  |  |  |  |
| S.E.M.: 804.339081212558 |  |  |  |  |  |
| S.E.D.: 1137.50723739751 |  |  |  |  |  |
| LSD (p<0.05): 2291.05718171882 | |  |  |  |  |
| LSD (p<0.01): 3059.42242513459 | |  |  |  |  |
