# Supplementary material for: Response of glyphosate-resistant and susceptible biotypes of Echinochloa colona to low doses of glyphosate in different soil moisture conditions
Source: PLoS One. 2020 May 20;15(5):e0233428. doi: 10.1371/journal.pone.0233428 (PMC7239466; doi:10.1371/journal.pone.0233428)
Supplement: S19 Table — (DOCX) [file pone.0233428.s021.docx]

| Table 19. ANOVA on glyphosate doses and water levels on plant height of glyphosate-resistant and susceptible biotypes of *Echinochloa colona* data in study ΙΙ | | | | | | | | | | | |
| --- | --- | --- | --- | --- | --- | --- | --- | --- | --- | --- | --- |
| **EFFECT** | **SS** | **DF** | **MS** | **F** | **ProbF** | **Sign. F** | **C.V. (%)** | **S.E.M.** | **S.E.D** | **L.S.D. (P<0.05)** | **L.S.D. (P<0.01)** |
| Replications | 11.70972222 | 5 | 2.341944444 | 0.222292404 | 0.952275896 |  |  |  |  |  |  |
| populations | 39.27111111 | 1 | 39.27111111 | 3.727530656 | 0.055984377 |  |  | 0.382524921 | 0.540971932 | 1.071561281 | 1.416945049 |
| water | 1922.8225 | 1 | 1922.8225 | 182.5102375 | 1.73066E-25 | ** |  | 0.382524921 | 0.540971932 | 1.071561281 | 1.416945049 |
| treatments | 24456.54306 | 5 | 4891.308611 | 464.2726493 | 1.78393E-74 | ** |  | 0.662552599 | 0.936990871 | 1.855998583 | 2.454220816 |
| populations x water | 13.56694444 | 1 | 13.56694444 | 1.28774562 | 0.258823568 |  |  | 0.540971932 | 0.765049842 | 1.515416497 | 2.003862905 |
| populations x treatment | 3498.318056 | 5 | 699.6636111 | 66.41058747 | 2.71941E-32 | ** |  | 0.936990871 | 1.325105197 | 2.624778368 | 3.470792364 |
| water x treatment | 569.1416667 | 5 | 113.8283333 | 10.80434421 | 1.56211E-08 | ** |  | 0.936990871 | 1.325105197 | 2.624778368 | 3.470792364 |
| populations x water x treatment | 58.88888889 | 5 | 11.77777778 | 1.117921711 | 0.35482357 |  |  | 1.325105197 | 1.873981742 | 3.711997166 | 4.908441633 |
| Residual | 1211.573611 | 115 | 10.53542271 |  |  |  | 8.115706154 |  |  |  |  |
| Total | 31781.83556 | 143 |  |  |  |  |  |  |  |  |  |
