# Supplementary material for: Response of glyphosate-resistant and susceptible biotypes of Echinochloa colona to low doses of glyphosate in different soil moisture conditions
Source: PLoS One. 2020 May 20;15(5):e0233428. doi: 10.1371/journal.pone.0233428 (PMC7239466; doi:10.1371/journal.pone.0233428)
Supplement: S20 Table — (DOCX) [file pone.0233428.s022.docx]

| Table 20. ANOVA on glyphosate doses and water levels on number of leaves per plant in the glyphosate-resistant and susceptible biotypes of *Echinochloa colona* data in study ΙΙ | | | | | | | | | | | |
| --- | --- | --- | --- | --- | --- | --- | --- | --- | --- | --- | --- |
| **EFFECT** | **SS** | **DF** | **MS** | **F** | **ProbF** | **Sign. F** | **C.V. (%)** | **S.E.M.** | **S.E.D** | **L.S.D. (P<0.05)** | **L.S.D. (P<0.01)** |
| Replications | 1176.618056 | 5 | 235.3236111 | 0.550632047 | 0.737547058 |  |  |  |  |  |  |
| populations | 82.50694444 | 1 | 82.50694444 | 0.193057413 | 0.661208577 |  |  | 2.436328031 | 3.445488144 | 6.824848899 | 9.02462232 |
| water | 30712.5625 | 1 | 30712.5625 | 71.8641069 | 8.90817E-14 | ** |  | 2.436328031 | 3.445488144 | 6.824848899 | 9.02462232 |
| treatments | 122674.7847 | 5 | 24534.95694 | 57.40917153 | 1.14581E-29 | ** |  | 4.219843934 | 5.967760523 | 11.82098505 | 15.63110438 |
| populations x water | 19.50694444 | 1 | 19.50694444 | 0.045644161 | 0.831202266 |  |  | 3.445488144 | 4.872656063 | 9.651793874 | 12.76274328 |
| populations x treatment | 6361.451389 | 5 | 1272.290278 | 2.977022986 | 0.014520934 | * |  | 5.967760523 | 8.439687869 | 16.71739737 | 22.1057198 |
| water x treatment | 8753.229167 | 5 | 1750.645833 | 4.096323754 | 0.001849165 | ** |  | 5.967760523 | 8.439687869 | 16.71739737 | 22.1057198 |
| populations x water x treatment | 552.9513889 | 5 | 110.5902778 | 0.258769406 | 0.934591221 |  |  | 8.439687869 | 11.93552105 | 23.6419701 | 31.26220875 |
| Residual | 49147.54861 | 115 | 427.3699879 |  |  |  | 27.40909453 |  |  |  |  |
| Total | 219481.1597 | 143 |  |  |  |  |  |  |  |  |  |
