# Supplementary material for: Response of glyphosate-resistant and susceptible biotypes of Echinochloa colona to low doses of glyphosate in different soil moisture conditions
Source: PLoS One. 2020 May 20;15(5):e0233428. doi: 10.1371/journal.pone.0233428 (PMC7239466; doi:10.1371/journal.pone.0233428)
Supplement: S21 Table — (DOCX) [file pone.0233428.s023.docx]

| Table 21. ANOVA on glyphosate doses and water levels on number of tillers per plant in the glyphosate-resistant and susceptible biotypes of *Echinochloa colona* data in study ΙΙ | | | | | | | | | | | |
| --- | --- | --- | --- | --- | --- | --- | --- | --- | --- | --- | --- |
| **EFFECT** | **SS** | **DF** | **MS** | **F** | **ProbF** | **Sign. F** | **C.V. (%)** | **S.E.M.** | **S.E.D** | **L.S.D. (P<0.05)** | **L.S.D. (P<0.01)** |
| Replications | 24.08333333 | 5 | 4.816666667 | 0.895460057 | 0.486713914 |  |  |  |  |  |  |
| populations | 196 | 1 | 196 | 36.4380978 | 1.975E-08 | ** |  | 0.273327884 | 0.386544 | 0.765669271 | 1.012458457 |
| water | 860.4444444 | 1 | 860.4444444 | 159.9640756 | 1.64969E-23 | ** |  | 0.273327884 | 0.386544 | 0.765669271 | 1.012458457 |
| treatments | 11672 | 5 | 2334.4 | 433.9851812 | 7.10876E-73 | ** |  | 0.473417782 | 0.669513848 | 1.326178078 | 1.753629489 |
| populations x water | 6.25 | 1 | 6.25 | 1.161929139 | 0.283321689 |  |  | 0.386544 | 0.546655768 | 1.082819867 | 1.431832482 |
| populations x treatment | 323.4166667 | 5 | 64.68333333 | 12.02519197 | 2.24915E-09 | ** |  | 0.669513848 | 0.946835564 | 1.875499025 | 2.480006606 |
| water x treatment | 221.6388889 | 5 | 44.32777778 | 8.240917868 | 1.10958E-06 | ** |  | 0.669513848 | 0.946835564 | 1.875499025 | 2.480006606 |
| populations x water x treatment | 17.33333333 | 5 | 3.466666667 | 0.644483363 | 0.66622599 |  |  | 0.946835564 | 1.339027695 | 2.652356157 | 3.507258978 |
| Residual | 618.5833333 | 115 | 5.378985507 |  |  |  | 11.24491637 |  |  |  |  |
| Total | 13939.75 | 143 |  |  |  |  |  |  |  |  |  |
