# Supplementary material for: Response of glyphosate-resistant and susceptible biotypes of Echinochloa colona to low doses of glyphosate in different soil moisture conditions
Source: PLoS One. 2020 May 20;15(5):e0233428. doi: 10.1371/journal.pone.0233428 (PMC7239466; doi:10.1371/journal.pone.0233428)
Supplement: S22 Table — (DOCX) [file pone.0233428.s024.docx]

| Table 22. ANOVA on glyphosate doses and water levels on number of inflorescence per plant in the glyphosate-resistant and susceptible biotypes of *Echinochloa colona* data in study ΙΙ | | | | | | | | | | | |
| --- | --- | --- | --- | --- | --- | --- | --- | --- | --- | --- | --- |
| **EFFECT** | **SS** | **DF** | **MS** | **F** | **ProbF** | **Sign. F** | **C.V. (%)** | **S.E.M.** | **S.E.D** | **L.S.D. (P<0.05)** | **L.S.D. (P<0.01)** |
| Replication | 82.08333333 | 5 | 16.41666667 | 2.678212555 | 0.025025268 | * |  |  |  |  |  |
| Populations | 6724 | 1 | 6724 | 1096.952358 | 1.20674E-60 | ** |  | 0.291778791 | 0.412637524 | 0.817355519 | 1.080804128 |
| water | 8464 | 1 | 8464 | 1380.815699 | 6.64271E-66 | ** |  | 0.291778791 | 0.412637524 | 0.817355519 | 1.080804128 |
| treatment | 31792.5 | 5 | 6358.5 | 1037.324743 | 7.11935E-94 | ** |  | 0.505375691 | 0.714709157 | 1.415701287 | 1.872007663 |
| Populations x water | 81 | 1 | 81 | 13.21432793 | 0.000416894 | ** |  | 0.412637524 | 0.583557583 | 1.155915261 | 1.528487856 |
| Populations x treatment | 1839.5 | 5 | 367.9 | 60.0191512 | 1.85948E-30 | ** |  | 0.714709157 | 1.010751383 | 2.002103961 | 2.647418625 |
| water x treatment | 1752.666667 | 5 | 350.5333333 | 57.18595579 | 1.34221E-29 | ** |  | 0.714709157 | 1.010751383 | 2.002103961 | 2.647418625 |
| Populations x water x treatment | 61.33333333 | 5 | 12.26666667 | 2.001182173 | 0.083632427 |  |  | 1.010751383 | 1.429418314 | 2.831402575 | 3.744015325 |
| Residual | 704.9166667 | 115 | 6.129710145 |  |  |  | 7.353936072 |  |  |  |  |
| Total | 51502 | 143 |  |  |  |  |  |  |  |  |  |
