# Supplementary material for: Response of glyphosate-resistant and susceptible biotypes of Echinochloa colona to low doses of glyphosate in different soil moisture conditions
Source: PLoS One. 2020 May 20;15(5):e0233428. doi: 10.1371/journal.pone.0233428 (PMC7239466; doi:10.1371/journal.pone.0233428)
Supplement: S23 Table — (DOCX) [file pone.0233428.s025.docx]

| Table 23. ANOVA on glyphosate doses and water levels on number of seed per plant in the glyphosate-resistant and susceptible biotypes of *Echinochloa colona* data in study ΙΙ | | | | | | | | | | | |
| --- | --- | --- | --- | --- | --- | --- | --- | --- | --- | --- | --- |
| **EFFECT** | **SS** | **DF** | **MS** | **F** | **ProbF** | **Sign. F** | **C.V. (%)** | **S.E.M.** | **S.E.D** | **L.S.D. (P<0.05)** | **L.S.D. (P<0.01)** |
| Replications | 9263235.004 | 5 | 1852647.001 | 1.460805886 | 0.208096937 |  |  |  |  |  |  |
| populations | 131279844.1 | 1 | 131279844.1 | 103.5137125 | 9.976E-18 | ** |  | 132.7192246 | 187.6933274 | 371.7843583 | 491.6172457 |
| water | 1944500577 | 1 | 1944500577 | 1533.23212 | 2.49829E-68 | ** |  | 132.7192246 | 187.6933274 | 371.7843583 | 491.6172457 |
| treatments | 3291274068 | 5 | 658254813.6 | 519.0316912 | 3.9359E-77 | ** |  | 229.8764401 | 325.0943793 | 643.9493981 | 851.5060475 |
| populations x water | 13407497.64 | 1 | 13407497.64 | 10.57176648 | 0.001506902 | ** |  | 187.6933274 | 265.4384492 | 525.7824818 | 695.2517764 |
| populations x treatment | 43697989.79 | 5 | 8739597.958 | 6.891143391 | 1.16477E-05 | ** |  | 325.0943793 | 459.7528802 | 910.6819722 | 1204.211401 |
| water x treatment | 556940317.9 | 5 | 111388063.6 | 87.82911088 | 1.29721E-37 | ** |  | 325.0943793 | 459.7528802 | 910.6819722 | 1204.211401 |
| populations x water x treatment | 16188466.42 | 5 | 3237693.284 | 2.552910189 | 0.031390442 | * |  | 459.7528802 | 650.1887586 | 1287.898796 | 1703.012095 |
| Residual | 145847170.5 | 115 | 1268236.265 |  |  |  | 11.14608824 |  |  |  |  |
| Total | 6152399167 | 143 |  |  |  |  |  |  |  |  |  |
