# Supplementary material for: Response of glyphosate-resistant and susceptible biotypes of Echinochloa colona to low doses of glyphosate in different soil moisture conditions
Source: PLoS One. 2020 May 20;15(5):e0233428. doi: 10.1371/journal.pone.0233428 (PMC7239466; doi:10.1371/journal.pone.0233428)
Supplement: S24 Table — (DOCX) [file pone.0233428.s026.docx]

| Table 24. ANOVA on glyphosate doses and water levels on plant biomass of glyphosate-resistant and susceptible biotypes of *Echinochloa colona* data in study ΙΙ | | | | | | | | | | | |
| --- | --- | --- | --- | --- | --- | --- | --- | --- | --- | --- | --- |
| **EFFECT** | **SS** | **DF** | **MS** | **F** | **ProbF** | **Sign. F** | **C.V. (%)** | **S.E.M.** | **S.E.D** | **L.S.D. (P<0.05)** | **L.S.D. (P<0.01)** |
| Replications | 12.85051458 | 5 | 2.570102917 | 0.809101931 | 0.545473509 |  |  |  |  |  |  |
| populations | 1.426034028 | 1 | 1.426034028 | 0.448934118 | 0.504184758 |  |  | 0.210042602 | 0.297045097 | 0.588389168 | 0.778037741 |
| water | 885.9056174 | 1 | 885.9056174 | 278.8946469 | 1.58442E-32 | ** |  | 0.210042602 | 0.297045097 | 0.588389168 | 0.778037741 |
| treatments | 1202.137881 | 5 | 240.4275763 | 75.6897379 | 9.75222E-35 | ** |  | 0.363804459 | 0.5144972 | 1.019119933 | 1.347600897 |
| populations x water | 0.662867361 | 1 | 0.662867361 | 0.208679294 | 0.648666226 |  |  | 0.297045097 | 0.420085205 | 0.832107941 | 1.100311525 |
| populations x treatment | 36.30602014 | 5 | 7.261204028 | 2.285921766 | 0.05065213 |  |  | 0.5144972 | 0.727608918 | 1.441253231 | 1.905795465 |
| water x treatment | 232.4969868 | 5 | 46.49939736 | 14.63861697 | 4.28996E-11 | ** |  | 0.5144972 | 0.727608918 | 1.441253231 | 1.905795465 |
| populations x water x treatment | 3.525803472 | 5 | 0.705160694 | 0.221993787 | 0.952410733 |  |  | 0.727608918 | 1.0289944 | 2.038239867 | 2.695201794 |
| Residual | 365.2961687 | 115 | 3.176488424 |  |  |  | 25.61883865 |  |  |  |  |
| Total | 2740.607894 | 143 |  |  |  |  |  |  |  |  |  |
